# Holistic frailty prevention: The promise of movement‐based mind–body therapies

**DOI:** 10.1111/acel.13986

**Published:** 2023-09-12

**Authors:** Julia Loewenthal, Michelle J. Berning, Peter M. Wayne, Elizabeth Eckstrom, Ariela R. Orkaby

**Affiliations:** ^1^ Division of Aging, Brigham and Women's Hospital Harvard Medical School Boston Massachusetts USA; ^2^ University of Minnesota Medical School Minneapolis Minnesota USA; ^3^ Division of Preventive Medicine Brigham and Women's Hospital Boston Massachusetts USA; ^4^ Osher Center for Integrative Medicine, Brigham and Women's Hospital Harvard Medical School Boston Massachusetts USA; ^5^ Division of General Internal Medicine & Geriatrics Oregon Health & Science University Portland Oregon USA; ^6^ New England Geriatric Research, Education, and Clinical Center (GRECC) VA Boston Healthcare System Boston Massachusetts USA

**Keywords:** aging, frailty, geroscience, mind–body, tai chi, yoga

## Abstract

Aging is characterized by fundamental cellular and molecular hallmarks that result in physiologic decline of most body systems. This may culminate in frailty, a state of decreased reserve. Because frailty is a state of multisystem dysregulation, multimodal interventions may be necessary to mitigate and prevent progression rather than interventions targeting a single system. Movement‐based mind–body therapies, such as tai chi and yoga, are promising multimodal strategies for frailty prevention and treatment given their inherent multicomponent nature. In this review, we summarize the links between hallmarks of aging and frailty and how tai chi and yoga may impact these hallmarks. We review trial evidence for the impact of tai chi and yoga on frailty in older populations and discuss opportunities for future research.

## Graphical abstract

Frailty is a state of multisystem dysregulation that is best addressed with multimodal interventions. Movement‐based mind–body therapies, like tai chi and yoga, are promising multimodal strategies for frailty given their multicomponent nature. Here, we summarize the effects of tai chi and yoga on the cellular and molecular hallmarks of aging and potential impacts on aging physiology and frailty.

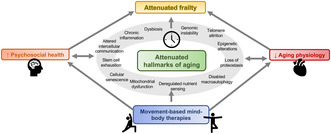

## 1 AGING AND FRAILTY

Aging, the time‐dependent decline in physiologic function that affects most living organisms, is characterized by 12 fundamental and related cellular and molecular “hallmarks”: genomic instability, telomere attrition, epigenetic alterations, loss of proteostasis, disabled macroautophagy, deregulated nutrient sensing, mitochondrial dysfunction, cellular senescence, stem cell exhaustion, altered intercellular communication, chronic inflammation, and dysbiosis (López‐Otín et al., 2023). As humans age, these cellular and molecular changes result in declines in physiologic systems. Frailty is characterized by a decline in functioning across multiple physiologic systems that is accompanied by increased vulnerability to stressors and increased morbidity and mortality (Clegg et al., 2013; Fried et al., 2001; Mitnitski et al., 2002). Risk factors for frailty include demographic and social, clinical, lifestyle, and biological factors (Hoogendijk et al., 2019). There is emerging evidence that biological mechanisms involved in the aging process increase susceptibility to frailty (Ferrucci et al., 2018).

Frailty is driven by the hallmarks of aging (Fried et al., 2021). If senescent cells are injected into younger mice physical frailty accelerates, whereas senolytic treatment reverses this trajectory (Xu et al., 2018). Mitochondrial dysfunction has a direct role in physical frailty, which has been demonstrated in mouse and human models (Akki et al., 2014; Andreux et al., 2018). In addition, frailty is linked to perturbations in multiple putative aging biomarkers, such as interleukin (IL)‐6, C‐reactive protein (CRP), tumor necrosis factor (TNF)‐α, insulin‐like growth factor (IGF)‐1, and others (Collerton et al., 2012; Gonçalves et al., 2022; Mitnitski et al., 2015). The Geroscience hypothesis posits that strategies targeted to modify these drivers of aging will prevent or delay the onset of multiple different chronic diseases (Burch et al., 2014; Kennedy et al., 2014; Sierra & Kohanski, 2013). Frailty is thought to emerge as a result of dysregulation in the complex dynamic system that is the human body (Fried et al., 2021); therefore, frailty models capturing multiple domains such as the Rockwood cumulative deficit model may offer a useful translational model to capture heterogeneity of aging (Howlett et al., 2021; Howlett & Rockwood, 2014).

There are two prevailing models that operationalize the clinical syndrome of frailty: the Fried physical phenotype (Fried et al., 2001) and Rockwood cumulative deficit model (Mitnitski et al., 2001, 2002). The Fried phenotype is characterized by five interrelated components: ≥10 lbs unintentional weight loss in the past year; self‐reported exhaustion; weakness measured by grip strength; slow walking speed; and decreased physical activity (Fried et al., 2001). Those with ≥3 components are considered frail. Rockwood and colleagues conceptualized frailty as an accumulation of health‐related deficits across multiple domains of health (e.g., morbidity, cognition, sensory impairment, and function) over the lifespan (Rockwood, 2016; Rockwood & Mitnitski, 2007; Searle et al., 2008). The total number of deficits for an individual are counted and divided by a total number of pre‐determined deficits to give a score between 0 and 1. Scores of 0.2–0.35 have been used to define frailty (Kim et al., 2018; Kulminski et al., 2008; Orkaby et al., 2019; Sheppard et al., 2014; Song et al., 2010). Scores above 0.7 are generally not observed in humans as further accumulated deficits most often result in death. Over 60 tools have been developed to measure frailty for both clinical and research purposes, and most derive from either the Fried or Rockwood approach (Ijaz et al., 2022).

The global prevalence of frailty among older adults is estimated to range from 12 to 24% depending on frailty classification (O'Caoimh et al., 2021). Systematic reviews have indicated that the prevalence of frailty ranges from 11% in community‐dwelling older adults to over 50% among long‐term care residents (Collard et al., 2012; Kojima, 2015). The prevalence of frailty is higher in women, people with lower socioeconomic status, and racial and ethnic minorities (Bandeen‐Roche et al., 2015; Hoogendijk et al., 2014; Santos‐Eggimann et al., 2009). Frailty is strongly associated with mortality, independent of age (Orkaby et al., 2019). In addition, frailty is associated with other adverse outcomes such as hospitalization and nursing home admission (Clegg et al., 2013).

Given the increasing prevalence of frailty with global aging and association with adverse outcomes, frailty prevention and management are priorities in clinical and public health. Interventions need to target multiple systems to be effective. Current evidence strongly supports physical activity, in particular multicomponent exercise, for both the prevention and reversal of frailty in older adults (Dent et al., 2019; Li et al., 2022; Theou et al., 2011). In addition, adherence to a Mediterranean style diet (Kojima et al., 2018; Talegawkar et al., 2012) and increased protein intake (Deer & Volpi, 2015; Fiatarone et al., 1994) appear to be effective. A recent network meta‐analysis compared effectiveness of nonpharmacological interventions for frailty, finding that physical activity was the most effective intervention; of note, mind–body exercise was reported to have a similar effect size to resistance training (pooled standardized mean difference [SMD] 0.57, 95% confidence interval [CI]: 0.24–0.90 vs. 0.58, 95% CI: 0.33–0.83), and was more effective than aerobic training alone (0.36, 95% CI: 0.09–0.62) (Sun et al., 2023). However, the four trials of mind–body exercise in this meta‐analysis only evaluated tai chi interventions and some only reported markers of frailty, such as the short physical performance battery (SPPB). Given their inherent complex systems approach, mind–body therapies, which uniquely integrate across multiple systems and thus potentially target multiple biological hallmarks of aging and frailty, warrant further investigation.

## 2 MIND–BODY THERAPIES ARE PROMISING APPROACHES TO FRAILTY PREVENTION AND MANAGEMENT

**FIGURE 1:**
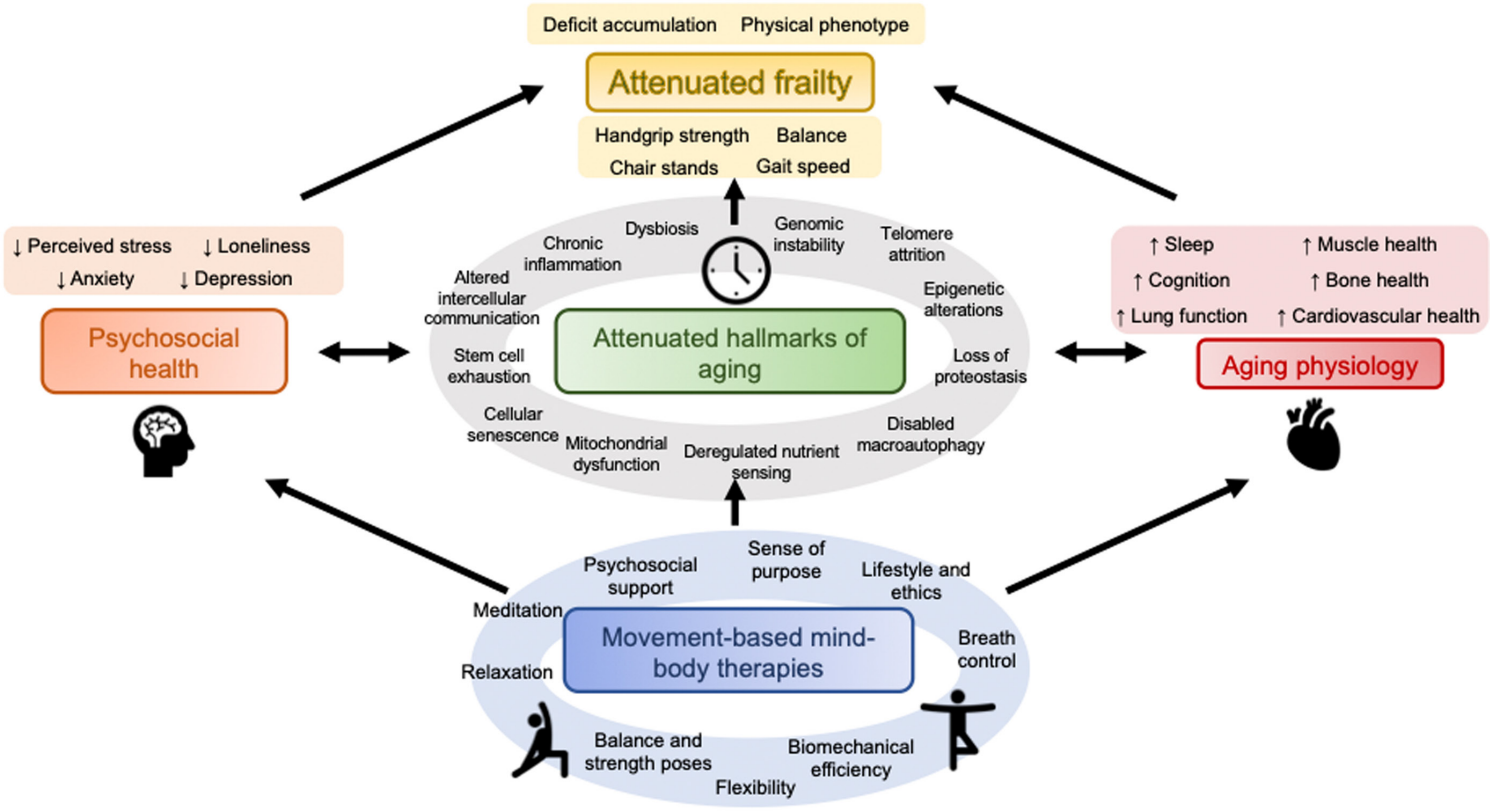
Conceptual model of putative frailty attenuation mechanisms. Movement‐based mind–body therapies (e.g., tai chi and yoga) incorporate multimodal practices that impact cellular and molecular hallmarks of aging, aging physiology, and psychosocial health of participants. These effects modulate frailty markers, with emerging evidence that they may improve frailty as measured by two prevailing models, physical phenotype and deficit accumulation.

Mind–body therapies include a variety of interventions such as movement‐based practices including tai chi, yoga, and qi gong as well as less physical practices, such as meditation, breath regulation, and relaxation. There is emerging evidence that mind–body therapies may impact certain hallmarks of aging. In a review of 26 randomized controlled trials (RCTs) of yoga, tai chi, qi gong, or meditation, there was decreased expression of inflammation‐related genes and reduced signaling through the proinflammatory transcription factor nuclear factor kappa B (NF‐κB) (Bower & Irwin, 2016). In a review of meditation randomized controlled trials, mindfulness meditation reduced activity of NF‐κB, reduced circulating CRP, increased CD4+ T cell count, and increased telomerase activity (Black & Slavich, 2016). In addition, mind–body therapies, including meditation, may impact epigenetic changes implicated in aging, including reduced methylation of TNF, altered expression of histone deacetylase, slower epigenetic clocks, and slower methylation of genes associated with inflammation (Kripalani et al., 2022). Movement‐based mind–body therapies are multimodal, coordinating not just breathing training and cognitive aspects, but also motor elements, making them promising strategies for frailty prevention and management. Here, we review how two movement‐based mind–body practices, tai chi and yoga, impact aging biology, physiologic systems, and frailty. Figure 1 provides a conceptual model of how these practices may support healthy aging and impact frailty.

### 2.1 Tai chi

Tai Chi, also referred to as Taiji, Tai Chi Chuan or Taijiquan, is a mind–body exercise that originated in China, and that is growing in popularity in the West. Tai Chi is based on slow intentional movements, often coordinated with breathing and imagery, that aims to strengthen and relax the physical body and mind, enhance the natural flow of “qi” (or life energy), and improve health, personal development, and in some systems, self‐defense (Lan et al., 2013). There are five main styles of Tai Chi, including Chen, Yang, Hao, Wu, and Sun that differ slightly from one another but share core training principles (“Tai Chi Forms,” 2023). There is no formal national or international certification process for teachers, so there may be heterogeneity in the content delivered by the instructors. A suggested minimum effective “dose” of tai chi from positive clinical trials is one to two 1‐h sessions per week for three to 12 months, supplemented with a modest home practice (Yang et al., 2021). Tai chi enhances physical health and quality of life among older adults, making it a strong intervention to promote healthy aging (de Souza Buto et al., 2020; Lee et al., 2022; Li et al., 2014). Among all United States (U.S.) adults, the prevalence of tai chi and qigong use increased from 1.2% (2.5 million) in 2007 to 1.7% (4.1 million) in 2017, and from 0.2% (343,000) to 0.5% (1.3 million) in adults age 65 and older (Wang et al., 2022). In a national sample of over 195,000 Australians, prevalence of tai chi and qigong use was 1.4% in adults 55 years and older in 2010 (Vergeer et al., 2017). In Shanghai, China, 22.4% of more than 130,000 middle age‐older adults reported tai chi use (Birdee et al., 2013). Studies show it is easily adaptable and modifiable for different abilities. Particularly for sedentary, deconditioned individuals starting an exercise program, tai chi has often been described as more accessible or less threatening and has been suggested as an alternative to other forms of conventional exercise or as a bridge to other physical activity (Fischer et al., 2014; Hart et al., 2004; Osypiuk et al., 2020; Taylor‐Piliae et al., 2012; Yeh et al., 2016).

At a cellular level, tai chi may have a protective effect on telomerase activity, which helps prevent telomere shortening and cellular aging (Duan et al., 2016; Hornsby, 2007). In addition, there is some evidence that tai chi alters DNA methylation to slow age‐related methylation losses or gains, suggesting it may impart beneficial epigenetic changes (Ren et al., 2012). Further studies have shown that tai chi impacts inflammatory markers involved in aging (Muñoz‐Vergara et al., 2023; You & Ogawa, 2019; You et al., 2020). In a three‐arm RCT of cognitive behavioral therapy vs. tai chi chih vs. sleep seminar, those in the tai chi arm had statistically significant reductions in proinflammatory cytokines and proinflammatory gene expression, as well as reduced activity of proinflammatory transcription factors (NF‐kB and activator protein 1) (Irwin et al., 2015). A systematic review of the impact of tai chi on inflammatory biomarkers found that tai chi resulted in statistically significant reductions in TNF‐α and IL‐6, but not CRP (Shu et al., 2021). Inflammatory markers are implicated in the development of frailty through mechanisms such as altered metabolic signaling, muscle cell apoptosis, and dysregulated tissue repair (Gonçalves et al., 2022; Hubbard & Woodhouse, 2010; Li et al., 2011). There is also evidence that tai chi may impact mitochondrial function by increasing antioxidant capacity (e.g., superoxide dismutase) and decreasing oxidative stress factors (e.g., plasma 8‐isoprostane and malondialdehyde) (Kasim et al., 2020; Liu, Salem, & Aggarwal, 2022). Mitochondrial function is directly implicated in the development of frailty as age‐related mitochondrial dysfunction limits muscle tissue regeneration, leading to loss of muscle mass and strength (Short et al., 2005). This loss contributes to multiple physical frailty phenotype criteria, including weak grip strength, slow gait speed, and low energy expenditure (Ness et al., 2015). Taken together, this research offers insight into how tai chi may impact several hallmarks of aging and potentially prevent the development of frailty.

**FIGURE 2:**
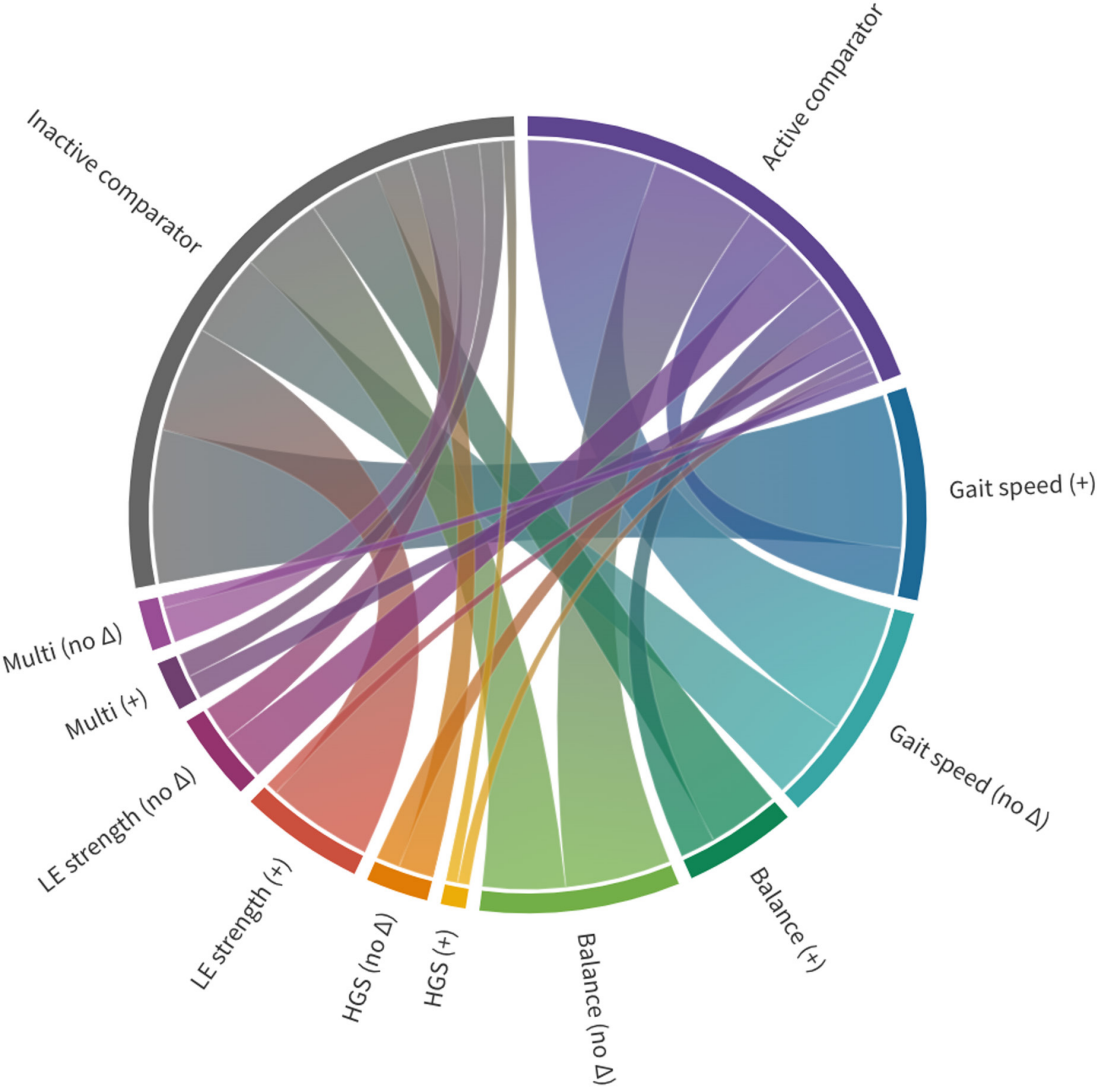
Chord diagram displaying results of 35 randomized controlled trials (RCTs) of a movement‐based mind–body therapy (9 tai chi and 26 yoga) with select frailty outcomes. “Active comparator” denotes RCTs with exercise or other active control group; “inactive comparator” denotes RCTs with education, waitlist, or usual care control groups. Frailty outcomes include gait speed, balance, handgrip strength (HGS), lower extremity (LE) strength and endurance, and multicomponent (“multi”) measures (e.g., Short Physical Performance Battery [SPPB]). “+” indicates a statistically significant between‐group improvement in the indicated outcome whereas “no Δ” indicates that there was no difference between groups. The thickness of each “chord” indicates the number of studies with the outcome.

Tai chi targets multiple components of health by improving general physical performance, balance, postural stability, flexibility, and lower limb strength (Huang & Liu, 2015; Li et al., 2005; Woolford et al., 2020). Previous studies have evaluated the impact of tai chi on validated markers of frailty, including gait speed (Lee et al., 2022), handgrip strength (HGS) (Leong et al., 2015), balance (Dayhoff et al., 1998), and chair stands (30‐s chair stand test [30CST]) (Millor et al., 2014). A systematic review and meta‐analysis of 11 clinical trials demonstrated statistically significant improvements in physical performance (30CST, timed up and go test [TUG]), as well as reduced number of falls and fear of falling among older adults with frailty or sarcopenia in the tai chi intervention group compared to the control group (Huang et al., 2022). Eight studies from this systematic review and other high‐quality RCTs identified from PubMed search performed in January 2023 (“tai chi” [AND] “frailty”) are summarized in Table 1 and Figure 2. In terms of mechanisms, there is evidence that tai chi enhances physiologic complexity of standing postural control, measured with standing center‐of‐pressure dynamics (Manor et al., 2013). There is also evidence supporting the combination of tai chi with other exercise interventions for frailty management (Liu, Wang, et al., 2022; Meng et al., 2022). In a three‐arm RCT with 150 frail older adults (mean age 76.3 years), 45% of frail older adults who were randomized to a 24‐week hybrid exercise intervention (Tai Chi + strength/endurance exercise) reversed from a frail to non‐frail phenotype, compared to 35% of participants in the strength/endurance group and 20% of the tai chi group. At 24 weeks, the hybrid exercise group had the largest increase in gait speed compared to the strength/endurance and tai chi groups. Grip strength increased in all groups, with the most significant improvements in the strength/endurance exercise and hybrid exercise groups (Meng et al., 2022).

**TABLE 1:** Summary of nine studies comparing the impact of tai chi on frailty markers in older adult populations.

References	Population (mean age [SD])	Intervention	Comparator	Main findings
Dechamps et al. (2009)	Older adults with frailty in nursing home setting (*n* = 52); mean age: 80.8 (8.7); 69.2% female.	Yang‐style Tai Chi; 30 min, 4×/week, 24 weeks	“Cognition‐action” exercise program; 30 min, 2×/week, 24 weeks	No difference in TUG or balance at 24 weeks
Ge et al. (2022)	Older adults with pre‐frailty in senior living setting (*n* = 65); mean age: 70.16 (5.40); 34.4% female.	8‐form Yang Style Tai Chi; 60 min, 3×/week, 8 weeks	Usual care: normal daily activities (e.g., art, bingo, seminars on aging, outings to movies)	Improved chair stand* and gait speed* at 8 weeks
Jiayuan et al. (2022)	Older adults with pre‐frailty and frailty (*n* = 93); mean age: 71.3 (5.0); 55.2% female.	Mindfulness‐based Tai Chi Chuan (24‐form Yang‐Style Tai Chi and mindfulness practice); 60 min, 2×/week, 6 months	(1) Tai Chi; 60 min, 2×/week, 6 months (2) Mindfulness practice; 60 min, 2×/week, 6 months	Improved SPPB* and TUG* at 6 months and 12 months (mindfulness‐based tai chi vs. tai chi vs. mindfulness) Improved Fried frailty phenotype* at 12 months
Kasim et al. (2020)	Older adults with frailty (*n* = 21), mean age: 71 (3.1); 63.6% female.	18‐form Yang‐style Tai Chi; 60 min, 3×/week, 12 weeks	Zumba Gold; 60 min, 3×/week, 12 weeks	No difference in TUG at 12 weeks
Liu, Wang, et al. (2022)	Older adults with frailty in nursing home setting (*n* = 135); mean age: 80.75 (2.99); 76.1% female.	Tai Chi and aerobic exercise; 40 min, 5×/week, 12 months	Usual Care: normal daily activities	Improved Fried frailty phenotype* and gait speed* at 12 months
Meng et al. (2022)	Older adults with frailty (*n* = 150); mean age: 76.31 (2.07); 60% female.	8‐form Tai Chi and strength/endurance exercise; 60 min, 3×/week, 24 weeks	(1) 8‐form Yang‐style Tai Chi; 60 min, 3×/week, 24 weeks (2) Strength/endurance exercise; 60 min, 3×/week, 24 weeks	Improved gait speed,* no difference in TUG at 24 weeks (Tai Chi + exercise vs. Tai Chi vs. exercise) Improved HGS* at 24 weeks (exercise vs. tai chi + exercise vs. tai chi) 69 (46.0%) improved from frail to non‐frail (44.9% tai chi + exercise, 34.8% exercise, 20.3% Tai Chi)
Morawin et al. (2021)	Older adults with sarcopenia (*n* = 80); mean age: 70.5 (5.8); 90% female.	24‐form Yang‐style Tai Chi; 40 min, 2×/week, 10 months	Health education program: lectures and discussions with a physician, dietician, or psychologist. 40 min, 2×/week, 10 months	No difference in gait speed or HGS at 10 months
Wolf et al. (2006)	Older adults with frailty (*n* = 286); mean age: 81.0 (6.4); 94% female.	6‐form Yang‐style Tai Chi; 60 min, 2×/week, 12 months	Wellness education: lectures on aging, fall prevention, exercise/balance, diet/nutrition, mental health. 60 min, 1×/week, 12 months	Improved chair stand,* no difference in gait speed at 12 months
Zhu et al. (2019)	Older men with sarcopenia (*n* = 79); mean age: 88.8 (3.7); 0% female.	8‐form Yang‐style Tai Chi; 40 min, 5×/week, 8 weeks	(1) Vibration therapy; 40 min, 5×/week, 8 weeks (2) Usual care: Normal daily activities. Participants received reminders to not change their level of physical exercise.	Improved physical performance (balance, gait speed, TUG, chair stand)* at 8 weeks (tai chi vs. usual care) No difference in TUG, chair stand, or gait speed at 8 weeks (tai chi vs. vibration therapy) No difference in HGS at 8 weeks (tai chi vs. vibration therapy or usual care)

*Note*: Mean age (standard deviation or range) and % female sex reported for tai chi group. “Education” indicates control group received non‐exercise education and is described when reported by study authors. “Waitlist control” indicates control group was assigned to a waitlist to receive the intervention after trial completion. “Usual care” indicates the control group was asked to continue their usual activities.

Abbreviations: HGS, handgrip strength; TUG, Timed Up and Go test.

* *p* ≤ 0.05.

In addition to improving frailty, there is also evidence that tai chi may prevent progression to frailty in non‐frail and pre‐frail older adults. A prospective cohort study including 5979 older adults (mean age 66.6 years) demonstrated that frequent tai chi involvement, defined as at least one session per week, was associated with decreased incidence of pre‐frailty or frailty among robust older adults (adjusted odds ratio [OR] = 0.41, 95% CI 0.19–0.80) (Lee et al., 2022). Similarly, an RCT investigating a tai chi intervention in 65 pre‐frail older adults (mean age 70.2 years) reported statistically significant improvements in walking speed, 30CST, fear of falling, and depression in the tai chi group compared to the control group (Ge et al., 2022). This emerging evidence supports the promising role of tai chi for prevention and management of frailty.

Importantly, tai chi is often accessible to older adults, either through Medicare Advantage programs (e.g., Silver Sneakers, which offers in person and online tai chi classes) or senior and community centers (e.g., Tai Ji Quan: Moving for Better Balance and Tai Chi for Arthritis) for free or minimal class fees.

### 2.2 Yoga

Yoga is believed to have originated in India around 5000 BC and consists of multiple elements: ethics (*yamas*), positive habit patterns (*niyamas*), posture (*asana*), voluntary control of breathing (*pranayama*), relaxation and sense withdrawal (*pratyahara*), concentration (*dharana*), meditation (*dhyana*), and enlightenment (*samadhi*) (Khalsa et al., 2016). The term “yoga” is derived from Sanskrit and means “to unite” or “to yoke” the body, mind, and spirit with the goal of spiritual liberation. Introductory points into yoga are most commonly physical postures, breathing practices, and relaxation skills. In modern times, yoga is usually associated with physical postures and includes a variety of traditions, schools, and styles (Wang et al., 2019). Classes are typically taught by an instructor, ideally with formal training and certification, in a group setting, but are increasingly offered in virtual or online formats. Yoga classes usually consist of physical postures in standing, seated, lying (supine and prone), and inverted positions. Coordinated breathing is emphasized and standalone breathing practice (*pranayama*) may also be offered as a component of the class. Focus is directed on integrating all elements of practice, including movement, breathing, and a gazing point (*drishti*), fostering a meditative state. Meditation may also be offered as a distinct part of practice. A suggested effective “dose” yoga practice for clinical outcomes is two to three 1‐h sessions per week with a home practice (Ross et al., 2012; Uebelacker et al., 2010). Yoga is increasingly popular: among all U.S. adults, yoga use increased from 9.5% in 2012 to 14.3% in 2017, and in adults age 65 and older from 3.3% to 6.7% (Clarke et al., 2018). In a 2014 survey, 19.4% of the German population reported yoga practice (Cramer, 2015) while 6.8% of Australian women aged 53–95 years with chronic conditions reported yoga use (Lauche et al., 2019). In India, 11.8% of more than 100,000 respondents reported yoga use (Mishra et al., 2020).

Yoga may impact cellular and molecular hallmarks of aging. In rodents, stretching protocols similar to yogic stretching seemed to positively impact inflammation (Muñoz‐Vergara, Grabowska, et al., 2022). In clinical studies, yoga has been shown to reduce stress‐related cellular aging, increase telomerase activity, and preserve telomere length (Krishna et al., 2015; Lavretsky et al., 2013). After a 3‐month yoga retreat, participants (mean age 34.8 years) were found to have increased brain‐derived neurotrophic factor (BDNF) and improved inflammatory biomarkers (reduced IL‐12 and increased IL‐10), though IL‐6, a proinflammatory cytokine, increased (Cahn et al., 2017). However, a trial of a 10‐week yoga intervention in depressed individuals (mean age 45.2 years) reported decreased IL‐6 with no changes in CRP or TNF‐α (Nugent et al., 2021). After a similar yoga intervention, participants (mean age 40.3 years) had statistically significant improvements in a marker of DNA damage deoxyguanosine, reactive oxygen species, cortisol, IL‐6, sirtuin‐1, and BDNF, among other markers of cellular aging (Tolahunase et al., 2017). In an RCT of an 8‐week yoga intervention in chronically stressed women (mean age 41.1 years), there was reduced TNF methylation in the yoga group but no significant differences in markers of inflammation or DNA methylation (Harkess et al., 2016). Results may conflict due to differences in study design, inflammatory marker measurement, and non‐linear dynamics (e.g., IL‐6 increases acutely after exercise and then decreases) (Muñoz‐Vergara, Schreiber, et al., 2022). Overall, yoga interventions appear to reduce some inflammatory biomarkers, but the full impact of yoga on the hallmarks of aging is not yet fully understood (Djalilova et al., 2019; Falkenberg et al., 2018).

There is evidence that yoga impacts intermediate physiologic systems between cellular and molecular changes and organ systems. Yoga practitioners have been observed to self‐regulate autonomic nervous system function, heart rate, and respiration (Wenger & Bagchi, 1961). Yoga practice is known to promote parasympathetic tone and decrease sympathetic response by mechanisms such as direct stimulation of vagal afferents and rhythmic breathing practices (Innes et al., 2007). In addition, yoga modulates the hypothalamic–pituitary–adrenal axis (HPA), reducing markers such as cortisol, catecholamines, and renin–angiotensin (Purdy, 2013; Riley & Park, 2015). Sympathetic activation and HPA dysregulation contribute to insulin resistance and impaired glucose metabolism, implicated in frailty (Fried et al., 2021). Yoga practices promote improvements in glycemic control, body composition, and lipid profiles (Innes & Selfe, 2016). Movement‐based yoga practices enhance muscular strength and endurance, though generally at lower levels than aerobic exercise (Khalsa et al., 2016). While there are limited data on mitochondrial effects, in one trial of an eight‐week yoga intervention in 70 participants with rheumatoid arthritis (mean age 45 years), the yoga group was found to have improvements in markers of mitochondrial health (Gautam et al., 2021).

Yoga positively impacts multiple domains of physical and psychological health (Büssing et al., 2012), including improved cardiovascular risk factors (Chu et al., 2016; Innes & Selfe, 2016), pulmonary function (Abel et al., 2013), and cognition (Bhattacharyya et al., 2021; Chobe et al., 2020); and reduced perceived stress (Pascoe & Bauer, 2015), depression (Cramer et al., 2013), and anxiety (Cramer et al., 2018). In older adult populations, yoga has been shown to improve balance and mobility (Youkhana et al., 2016), physical function (Sivaramakrishnan et al., 2019), and mental well‐being and quality of life (Kelley & Kelley, 2020; Tulloch et al., 2018). While no controlled studies have specifically examined the effect of yoga on operational definitions of frailty (e.g., Fried physical phenotype or Rockwood cumulative deficit), multiple studies have included validated markers of frailty. In a recent systematic review, we examined the evidence of yoga practice on these frailty markers, finding moderate‐certainty evidence that yoga improves gait speed and chair stands, as compared to inactive control groups (Loewenthal et al., 2023). We summarize 26 studies with low or some concerns of risk‐of‐bias, based on the Cochrane revised tool for assessing risk‐of‐bias in randomized trials in Table 2 and Figure 2 (Sterne et al., 2019). Studies of Iyengar yoga tended to result in greater improvements in gait speed. For example, Tiedemann et al., 2013 evaluated a 12‐week Iyengar yoga intervention in 54 community‐dwelling older adults (mean age 67.7 years) as compared to waitlist control, finding clinically meaningful and statistically significant improvements in gait speed and lower extremity strength and endurance between groups (Tiedemann et al., 2013). In a study of 135 healthy older adults (mean age 71.5 years), 6 months of weekly Iyengar yoga resulted in clinically meaningful and statistically significant improvement in gait speed, even when compared to an exercise intervention. Regimens can be adapted for more frail participants, such as Gentle Years Yoga, which makes poses more accessible for older adults with physical disability or cognitive impairment, or Sit “N” Fit Chair Yoga, which is performed entirely in a seated position (Park et al., 2017; Tew et al., 2017).

**TABLE 2:** Summary of 26 studies comparing the impact of yoga on frailty markers in older adult populations.

References	Population (mean age [SD])	Intervention	Comparator	Main findings
Bega et al. (2016)	Older adults with Parkinson's (*n* = 17); mean age: 67.9 (10.9); 28.5% female.	Iyengar yoga; 60 min, 2×/week, 12 weeks; no home practice.	Resistance training; 60 min, 2×/week, 12 weeks.	No difference in gait speed or balance at 12 weeks.
Čekanauskaitė et al. (2020)	Physically inactive older adults (*n* = 33); mean age: 66.9 (6.0); 91% female.	Yoga; 90 min, 2×/week, 10 weeks; no home practice.	Usual care	Improved balance* at 10 weeks.
Chen et al. (2008)	Older adults at senior centers (*n* = 204); mean age: 68.9 (6.3); 72.7% female.	Silver yoga with and without meditation; 70 min, 3×/week, 24 weeks; no home practice.	Waitlist	Improved gait speed* and LE strength/endurance* at 12 and 24 weeks.
Cherup et al. (2021)	Older adults with Parkinson's (*n* = 46); mean age: 69.8 (7.3); 33% female.	Yoga Meditation; 45 min, 2×/week, 12 weeks; no home practice.	Proprioceptive training; 45 min, 2×/week, 12 weeks; no home practice.	Improved balance,* no difference in gait speed at 12 weeks (yoga vs. proprioceptive training).
Cheung et al. (2017)	Older adults with knee OA (*n* = 83); mean age: 68.9 (7.7); 84% female.	Hatha yoga; 45 min, 1×/week, 8 weeks; home practice.	(1) Aerobic and strengthening exercises; 45 min, 1×/week, 8 weeks; home practice. (2) Education (printed OA brochures, weekly phone calls).	No difference in gait speed, balance, LE strength/endurance, or SPPB at 8 weeks (yoga vs. exercise). Improved gait speed* and LE strength/endurance*, no difference in balance or SPPB at 8 weeks (yoga vs. education).
Cheung, Wyman, Resnick, & Savik (2014)	Older women with knee OA (*n* = 36); mean age: 71.9 (69.3 to 74.6); 100% female.	Yoga; 60 min, 1×/week, 8 weeks; home practice.	Waitlist	Improved LE strength/endurance* and SPPB,* no difference in gait speed or balance at 8 weeks.
Donesky‐Cuenco et al. (2009)	Older adults with COPD (*n* = 41); mean age: 72.2 (6.5); 71% female.	Iyengar yoga; 60 min, 2×/week, 12 weeks; daily home practice.	Waitlist (received COPD educational pamphlet)	Improved gait speed* at 12 weeks.
Greendale et al. (2009)	Older adults with adult‐onset hyperkyphosis (*n* = 118); mean age: 74.5 (7.6); 82.8% female.	Hatha yoga; 60 min, 3×/week, 24 weeks; no home practice.	Education (health seminars); 120 min, 1×/month, 24 weeks.	No difference in gait speed or LE strength/endurance at 24 weeks.
Groessl et al. (2018)	Physically inactive older adults (*n* = 46); mean age: 71.6 (8.3); 68% female.	Silver Age Yoga; 60 min, 2×/week, 10 weeks; home practice.	Education (health information); 90 min, 1×/week, 10 weeks.	No difference in gait speed, HGS, balance, LE strength/endurance, or SPPB at 10 weeks
Khuzema et al. (2020)	Older adults with Parkinson's (*n* = 27); mean age 68.1 (4.2); 33% female.	Yoga; 30–40 min, 1 session; 5 days/week home practice; 8 weeks.	(1) Tai chi; 30–40 min, 1 session; home practice 5 days/week; 8 weeks. (2) Balance exercise; 40–45 min, 1 session.	No difference in gait speed or balance at 8 weeks.
Marques et al. (2017)	Nursing home (*n* = 47); mean age 83.7 (6.9); 100% female.	Chair yoga; 50 min, 2‐3×/week, 28 weeks; no home practice.	Usual care	No difference in gait speed at 28 weeks.
McCaffrey et al. (2019)	Older adults with OA (*n* = 18); mean age 79 (2.5); 75% female.	Chair yoga; 50 min, 2×/week, 8 weeks; no home practice.	Chair exercise; 50 min, 2×/week, 8 weeks.	No difference in gait speed at 8 weeks.
Milbury et al. (2019)	Older adults with lung or esophageal cancer undergoing radiotherapy (*n* = 52); mean age: 66.2 (5.5); 38% female.	Dyadic yoga (with caregiver); 60 min, 2‐3×/week, 6 weeks; home practice.	Waitlist	Improved gait speed* at 6 weeks
Ni et al. (2014)	Community‐dwelling older adults with at least 1 fall in past year (*n* = 48); mean age: 73.2 (5.1); 77% female.	Balance yoga; 60 min, 2×/week, 12 weeks; no home practice.	(1) Tai chi; 60 min, 2×/week, 12 weeks; no home practice. (2) Standard balance exercise; 60 min, 2×/week, 12 weeks; no home practice.	No difference in gait speed or balance at 12 weeks (yoga vs. tai chi vs. standard balance exercise).
Ni et al. (2016)	Older adults with Parkinson's (*n* = 41); mean age: 71.2 (6.5); 15.4% female.	Power yoga; 60 min, 2×/week, 12 weeks; no home practice.	(1) Power training; 60 min, 2×/week, 12 weeks; no home practice. (2) Non‐exercise health education class; 60 min, 1×/month, 12 weeks.	Improved balance* and gait speed* at 12 weeks (yoga vs. education). No difference in balance or gait speed at 12 weeks (yoga vs. power training).
Nicholson et al. (2014)	Physically active older adults (*n* = 31); mean age: 66.0 (4.9); 73.3% female.	Body Balance, 2×/week, 12 weeks; no home practice.	Usual care	Improved gait speed* and LE strength/endurance at 12 weeks.
Nick et al. (2016)	Older adults with falls risk (*n* = 40); mean age: 68 (4.9); 55% female.	Yoga; 60 min, 2×/week, 8 weeks; no home practice.	Usual care	Improved balance* at 8 weeks.
Noradechanunt et al. (2017)	Physically inactive older adults (*n* = 39); mean age: 67.6 (4.9); 76.9% female.	Thai Yoga; 80 min, 2×/week, 12 weeks; home practice.	(1) Tai chi; 80 min, 2×/week, 12 weeks; home practice. (2) Education (exercise education advice).	Improved gait speed* and LE strength/endurance* at 24 weeks (yoga vs. tai chi) Improved gait speed* and LE strength/endurance* at 24 weeks (yoga vs. education)
Oken et al. (2006)	Healthy older adults (*n* = 135); mean age: 71.5 (4.9); 70.4% female.	Iyengar yoga; 90 min, 1×/week, 6 months; home practice.	(1) Exercise; 60 min, 1×/week, 6 months; home exercise. (2) Waitlist	Improved gait speed* and balance,* no difference in LE strength/endurance at 6 months (yoga vs. exercise vs. waitlist)
Park et al. (2017)	Older adults with OA (*n* = 131); mean age: 75.9 (8.2); 69.8% female.	Chair yoga; 45 min, 2×/week, 8 weeks; home practice.	Education about OA and exercise, 45 min, 2×/week, 8 weeks	Improved gait speed,* no difference in balance at 8 weeks.
Saravanakumar et al. (2014)	Nursing home (*n* = 33); mean age: 84.0 (6.7); 90.9% female.	Yoga; 30 min, 2×/week, 14 weeks; no home practice.	(1) Tai chi; 30 min, 2×/week, 14 weeks; no home practice. (2) Usual care (encouraged to attend exercise class)	No difference in balance at 14 weeks (yoga vs. tai chi vs. usual care)
Taskiran et al. (2014)	Nursing home (*n* = 58); mean age: 77.2 (6.4); 83.3% female	Yoga; 50 min, 3×/week, 8 weeks; no home practice.	(1) Pilates; 50 min, 3×/week, 8 weeks; no home practice. (2) Usual care	Improved HGS* at 8 weeks (yoga vs. usual care). No difference in gait speed, balance, LE strength/endurance, or HGS at 8 weeks (yoga vs. pilates vs. usual care).
Teut et al. (2016)	Older adults with chronic low back pain (*n* = 176); mean age: 73.0 (5.6); 88.5% female.	Viniyoga; 45 min, 2×/week, 12 weeks; no home practice.	(1) Qigong, 90 min, 1×/week, 12 weeks. (2) Waitlist	No difference in balance or HGS at 3 months (yoga vs. waitlist)
Tew et al. (2017)	Healthy older adults (*n* = 52); mean age 73.8 (6.5); 100% female.	Gentle Years Yoga; 75 min, 1×/week, 12 weeks; no home practice.	Waitlist	No difference in gait speed, balance, LE strength/endurance, or SPPB at 12 weeks
Tiedemann et al. (2013)	Community‐dwelling older adults (*n* = 54); mean age: 67.7 (7.2); 82% female.	Iyengar yoga; 60 min, 2×/week, 12 weeks; home practice.	Waitlist	Improved gait speed,* balance,* and LE strength/endurance* at 12 weeks
Van Puymbroeck et al. (2018)	Older adults with Parkinson's disease (*n* = 30); mean age: 65.53 (6.1); 33.3% female.	Yoga; 2×/week, 8 weeks; no home practice.	Waitlist	Improved FGA,* no difference in balance at 8 weeks
Wooten et al. (2018)	Older adults with falls (*n* = 30); mean age: 74.8 (9.6); 100% female.	Yoga Meditation; 45 min, 3×/week, 6 weeks; no home practice.	Proprioceptive training; 45 min, 3×/week, 6 weeks; no home practice.	No difference in balance at 6 weeks.
Zhu et al. (2021)	Community‐dwelling older adults (*n* = 54); mean age 66.8 (13.7); 44.4% female.	Iyengar yoga; 60 min, 4×/week, 16 weeks; no home practice.	(1) Tai chi; 60 min, 4×/week, 16 weeks; no home practice. (2) Usual care (walk outside 30 min 1–2× per week).	Improved gait speed* and balance* at 16 weeks (yoga vs. tai chi). Improved gait speed* and balance* at 16 weeks (yoga vs. usual care).

*Note*: Mean age and % female sex (standard deviation or range) reported for yoga group. “Education” indicates control group received non‐exercise education and is described when reported by study authors. “Waitlist control” indicates control group was assigned to a waitlist to receive the intervention after trial completion. “Usual care” indicates the control group was asked to continue their usual activities.

Abbreviations: COPD, chronic obstructive pulmonary disease; FGA, Functional Gait Assessment; HGS, handgrip strength; LE, lower extremity; OA, osteoarthritis; SPPB, short physical performance battery.

* *p* ≤ 0.05.

Older adults can access yoga classes, particularly chair yoga, through local senior centers for low to minimal cost. SilverSneakers offers yoga classes in a variety of formats, including online. Lists of registered yoga teachers can be accessed through Yoga Alliance. Yoga therapists have additional training and expertise in working with persons with medical conditions and can be located through the International Association of Yoga Therapists.

## 3 HEALTH DISPARITIES

Frailty is associated with lower socioeconomic status, with higher prevalence among older adults living in deprived neighborhoods, low education, and/or low income (Lang et al., 2009; Szanton et al., 2010). Disparities exist in integrative therapy use, with lower rates among non‐Hispanic blacks and Hispanics as compared to non‐Hispanic whites; adults with lower educational attainment; and poor individuals (Clarke et al., 2015; 2018). Factors impacting participation include awareness, availability, accessibility, and affordability (R. Saper, 2016). Yoga use is highest among those with higher socioeconomic status and women (Park et al., 2015). However, research indicates that yoga interventions have positive benefits and are acceptable to those with lower socioeconomic status, including racial and ethnic minorities (Keosaian et al., 2016; Middleton et al., 2017; Saper et al., 2009, 2013; Spadola et al., 2017). Among US Veterans, almost half of whom are ≥65 years (Farrell et al., 2023), 7.4% of those with chronic pain and 14.2% with both chronic pain and post‐traumatic stress disorder (PTSD) reported yoga use, which is freely available through Veterans Affairs medical centers. Similarly, 6.1% of Veterans with chronic pain and 10.3% with both chronic pain and PTSD reported tai chi use (Reed II et al., 2022). While U.S. older adults can access movement‐based mind–body therapies for reduced or no cost through senior centers, rates of yoga and tai chi use through these access points are unknown. A tai chi program (Tai Ji Quan: Moving for Better Balance) was successfully implemented through an Area Agency on Aging serving non‐English speaking older adults and multiple rural faith‐based organizations (Fink & Houston, 2014; Jones et al., 2016). Telehealth has improved access to yoga and tai chi, though services need to be adapted for older adults with technical literacy issues, cognitive impairment, and/or hearing loss (Hawley et al., 2020; Kruse et al., 2020; Murphy et al., 2020).

## 4 SEX DIFFERENCES

As compared with men, women tend to have a longer lifespan but higher burden of frailty (Hubbard, 2015). The exercise science literature has predominantly focused on men, with study populations containing only 36% female participants (Garver et al., 2023). Women may benefit more from physical activity than men; a systematic review of 17 studies reported greater risk reduction for incident stroke in women who were physically active (Madsen et al., 2022). Compared to men who are more likely to experience cardiovascular disease, women accumulate more musculoskeletal conditions such as sarcopenia and osteoporosis, suggesting that women may experience more benefit from resistance and balance training to enhance muscle strength and prevent falls (Reid et al., 2022). Sex differences may account for differences in training adaptation and muscle function between women and men, potentially influencing factors such as the optimal time of day to exercise (Ansdell et al., 2020; Beaven et al., 2014; Ives et al., 2017).

Movement‐based mind–body interventions variably report data by sex, and results are inconsistent. One study reported lower all‐cause mortality in 105 male and female tai chi practitioners, but this finding was significant only in men (Moriyama et al., 2023). Women tend to seek out and use integrative therapies more often than men. For instance, they are more than twice as likely to use yoga as compared with men (19.8% vs. 8.6%, use in past 12 months) and more likely to use meditation (10.3% vs. 5.2%, use in past 12 months) (Clarke et al., 2015, 2018). In addition, reasons for therapy use differ, with women reporting meditation use for general wellness and men to improve energy or performance (Upchurch & Johnson, 2019). Prior work in cardiac rehabilitation has shown that females who were less likely to participate in formal physical activity were more likely to use mind–body therapies (Leung et al., 2008; Thomas et al., 1996). Mind–body therapies may be an entry point to other types of physical activity for women; more work is needed to further characterize sex differences in preference, use of, and response to mind–body therapies.

## 5 SUMMARY AND FUTURE DIRECTIONS

Movement‐based mind–body therapies are promising holistic strategies for frailty prevention. Future clinical trials should incorporate operational definitions of frailty as outcomes, such as the physical phenotype and deficit accumulation models. Three tai chi studies included in this review included the Fried frailty phenotype as an outcome, but yoga studies typically targeted younger populations, only evaluated frailty markers and no studies used a deficit accumulation model (Table 1). In addition, complexity‐based physiologic measures, such as stride‐to‐stride gait variation, should be evaluated as they may be more sensitive to subtle decline than traditional measures (e.g., TUG) and may provide new insights into strategies for healthy aging (Wayne et al., 2013). Studies could evaluate an integrative “package” of practice as compared to its individual components, such as poses, breath regulation, meditation, and relaxation, to better understand how these elements interact. More research is needed in determining meaningful biomarkers to capture aging hallmarks, in addition to standardizing measurement, considering dynamics, and determining clinically meaningful changes. Future studies of movement‐based mind–body therapies and aging should consider incorporating biomarkers along with survey measures and clinical assessments. The frailty status of participants should be measured at trial enrollment since frailty may impact treatment effect (Quach et al., 2022). There are barriers to accessing mind–body therapies for older populations, including lack of insurance coverage, cost, and socioeconomic disparities (Saper, 2016); additional work in implementation is needed.

Tai chi and yoga are safe and effective, with well‐established evidence that they support physical and psychological health in older adult populations. This review summarizes the emerging evidence that movement‐based mind–body therapies positively impact multiple facets of aging, including cellular and molecular hallmarks.

## AUTHOR CONTRIBUTIONS

Julia Loewenthal and Ariela R. Orkaby were responsible for design. Julia Loewenthal and Michelle J. Berning were responsible for writing. Elizabeth Eckstrom, Peter M. Wayne, and Ariela R. Orkaby were responsible for editing. Julia Loewenthal generated figures and tables.

## FUNDING INFORMATION

Primary funding source: none. A.R.O. is supported by VA CSR&D CDA‐2 award IK2‐CX001800. J.L. is supported by HRSA GACA 6 K01HP49053‐01‐01. P.M.W. is supported by NIH K24 AT009282.

## CONFLICT OF INTEREST STATEMENT

P.M.W. is the founder and sole owner of the Tree of Life Tai Chi Center. Peter Wayne's interests were reviewed and managed by the Brigham and Women's Hospital and Partner's HealthCare in accordance with their conflict of interest policies. The authors have no other conflicts of interest to declare.

## Data Availability

Data sharing not applicable to this article as no datasets were generated or analysed during the current study
